# Role of the Angiotensin Pathway and its Target Therapy in Epilepsy Management

**DOI:** 10.3390/ijms20030726

**Published:** 2019-02-08

**Authors:** Shaip Krasniqi, Armond Daci

**Affiliations:** 1 Institute of Pharmacology and Toxicology, Faculty of Medicine, University of Prishtina, Prishtina 10000, Kosovo; shaip.krasniqi@uni-pr.edu; 2 Department of Pharmacy, Faculty of Medicine, University of Prishtina, Prishtina 10000, Kosovo

**Keywords:** renin–angiotensin system, epileptogenesis, antiepileptic drugs, personalized treatment

## Abstract

Despite extensive research on epileptogenesis, there is still a need to investigate new pathways and targeted therapeutic approaches in this complex process. Inflammation, oxidative stress, neurotoxicity, neural cell death, gliosis, and blood–brain barrier (BBB) dysfunction are the most common causes of epileptogenesis. Moreover, the renin–angiotensin system (RAS) affects the brain’s physiological and pathological conditions, including epilepsy and its consequences. While there are a variety of available pharmacotherapeutic approaches, information on new pathways is in high demand and the achievement of treatment goals is greatly desired. Therefore, targeting the RAS presents an interesting opportunity to better understand this process. This has been supported by preclinical studies, primarily based on RAS enzyme, receptor-inhibition, and selective agonists, which are characterized by pleiotropic properties. Although there are some antiepileptic drugs (AEDs) that interfere with RAS, the main targeted therapy of this pathway contributes in synergy with AEDs. However, the RAS-targeted treatment alone, or in combination with AEDs, requires clinical studies to contribute to, and clarify, the evidence on epilepsy management. There is also a genetic association between RAS and epilepsy, and an involvement of pharmacogenetics in RAS, so there are possibilities for the development of new diagnostic and personalized treatments for epilepsy.

## 1. Introduction

Epilepsy is a chronic neurological disorder of the central nervous system, characterized by abnormal, increased and persistent excitatory brain activity and synchronicity, which result in diverse disorders, such as recurrent seizures, loss of motor control, temporary confusion, unusual behavior, autonomic dysfunction, loss of consciousness, and cognitive or emotional symptoms, including, fear, anxiety and déjà vu [1]. Globally, epilepsy is considered to be one of the most important neurological diseases and one of the leading causes of disability-adjusted life years, with high rates of death, a lifetime prevalence rate of 6.4 per 1000 and an annual incidence rate of 61.4 per 100,000, depending on the developmental status of the country [2,3]. A detailed understanding of the pathophysiology remains incomplete, and there is not one single mechanism that clearly presents the diversity of causes and seizure types.

There are a variety of theories on epilepsy pathophysiology, including neurotransmitter misbalance and channelopathies, as well as causative factors, such as trauma, tumors, stroke, metabolic disorders, infections, inflammation, neural migration, neuronal loss, brain injuries, degenerative disorders, morphological abnormalities, cortical and/or hippocampal and hypothalamic malformations, blood–brain barrier dysfunction, and genetic background. Despite this, the causes and etiology of epilepsy are still unknown in about half of all those who are diagnosed with epilepsy [4,5,6,7].

Currently, the management of epilepsy is primarily based on an antiepileptic drug (AED) regimen, but despite its effectiveness, patients still exhibit a high percentage of pharmacoresistance (30–40%), which challenges clinicians and indicates a need for new therapeutic alternatives and a more serious approach to pharmacogenetics [8,9,10]. In spite of recent advances in molecular neuroimaging, pharmacogenetics and neuropathology, the current optimal treatment therapy for epilepsy is still significantly ineffective, implying a need for comprehensive research engagement and integration of the preclinical and clinical science, which may result in a more successful approach [11,12,13].

There has been a trend towards moving AED pharmacological research in several new directions, such as α-amino-3-hydroxy-5-methyl-4-isoxazolepropionic acid receptor-inhibition [14,15], protein kinase inhibition [16,17], metabotropic glutamate receptor subtype signaling function modulation [18,19], carbonic anhydrase inhibition [20,21], gamma amino butyric acid receptor modulation [22,23], the mammalian target of rapamycin inhibition [24,25], inflammation inhibition through targeting interlukin-1b [26,27,28], transforming growth factor beta (TGF-β) [29], drug transporter system improvement, including P-glycoprotein [30], the activation of hyperpolarization cyclic nucleotide gated channels [31], the opening of voltage-gated Kv7 channels [32], exploration of the role of nuclear-related factor 2 modulators [33], Na-K-2Cl, K-Cl co-transport modulators [34], purinergic-receptor modulation, and the role of aberrant neurogenesis in epilepsy and cannabidiol mechanism exploration in epilepsy [35,36,37,38]. These investigations are contributing to a comprehensive overview of epileptogenesis and targeted treatment mechanisms [39].

## 2. Role of Blood–Brain Barrier Dysfunction, Microglia, and Astrocyte Activation in Epilepsy Pathogenesis

Recently, studies on epilepsy and the epileptogenesis process have become more comprehensive, showing particular interest in BBB dysfunction, microglia, and astrocyte activation mechanisms [36,40]. The BBB is an important structure and a dynamic component in epileptogenesis, and comprises a microvessel basement membrane, endothelial cells, and tight junction proteins (TJPs), which are responsible for the impermeability of various pathogens and toxins, as well as astrocytes and pericytes [41]. Typically, the BBB ensures the homeostasis of the central nervous system (CNS), including nutrition delivery, ionic balance, immune-cell infiltration and vascular regulation [42]. Various physical injuries, exposure to pathogens, and inflammation trigger a response from these BBB structures, which then initiate sundry reactive activities, including an inflammatory response, increased expression of TJPs and activation of the compensatory mechanisms that maintain the primary function of the BBB [43].

In cases where physical injury or another pathological mechanism overcome these compensatory mechanisms, the integrity of the BBB may be threatened, resulting in increased permeability for different substances and agents, leading to an inflammatory response and neuronal hyperexcitability [44]. Therefore, BBB dysfunction contributes to the epileptogenesis process [45,46], and further dysfunction occurs due to traumatic brain injury, insults, particular infections, tumors, and febrile seizures in infants [47,48].

However, glia are constructed from microglial cells and astrocytes in the CNS, and are closely related to other structures in the brain and spinal cord [49,50]. Microglia are immunocompetent cells, localized in the brain tissue, that assemble in the immune network, and are activated in a primary protection response against microorganismal exposure and injuries so as to maintain homeostasis in a healthy brain and various CNS diseases [51]. Microglia have been identified as being important elements in brain maintenance, plasticity, protection, and the regulation of neurogenesis and oligodendrogenesis [52,53]. While microglial cells have a slower onset response in neuronal stimulation, they are involved in the long-term control of CNS functions through neuromodulation [54], clearance of apoptotic neurons [55], synaptogenesis [56], and amplified neuronal survival [57].

Astrocytes are located close to the neuronal synapses, acting as ion channels, neurotransmitter transporters and receptors, and are highly involved in neuronal activity, including learning and memory processing [58], sleep control [59], and breathing [60]. Moreover, they enable the structural and metabolic maintenance of synaptic neuronal connections, and mediate the equilibrium of extracellular concentrations of K^+^ through spatial K^+^-buffering processes in the glial–neuronal-coupling network and perform glutamate uptake, removing it from the extracellular space. In this latter process, the distribution of K^+^ channels, water purine channels and the gap junction in the astrocytes, as well as glutamate transport, glutamine synthase, and their functional changes, are triggered under pathological conditions in the astrocyte structures. This then produces neuronal hyperactivity, leading to the clinical manifestation of several diseases, including epilepsy.

Microglia, neurons, and astrocytes construct a dynamic environment that functions through highly-equilibrated, complex relationships [61,62]. Thus, it is well known that, in patients with temporal lobe epilepsy (TLE), microglia and astrocytes are activated, and inflammatory molecules and proinflammatory mediators migrate to the hippocampi [35,47], presenting a challenge to investigations aimed at understanding their main functional and metabolic relationships under pathological conditions, including brain damage and neuroinflammation [63,64]. The inflammation process also has a significant impact on epileptogenesis.

This is also apparent in the specific and important role of TGF-β signaling in epileptogenesis, following BBB dysfunction following a brain injury [65]. Under such conditions, different serum albumins permeate the BBB, bind to the TGF-β receptors and activate the astrocytes [66], which is considered to be a substantial mechanism in epileptogenesis following brain injury [67]. Activated astrocytes induce inflammatory pathway signaling, and several changes in the astrocytes and the surrounding neuronal environment are expressed, which may trigger epileptic seizures [68], associated with the upregulation of TGF-β expression in epileptogenesis [69]. Hence, there is great scientific interest in understanding the role of TGF-β in microglial cells [70].

Taking these findings into consideration, there is a suggestion that TGF-β-pathway exploitation may be a potential approach for the prevention of post-injury epilepsy [71]. Moreover, the inhibition of TGF-β signaling might be considered to be an additional therapeutic approach in epilepsy [29]. Therefore, elucidation of the renin–angiotensin system (RAS) effect in the inhibition of the TGF-β-pathway in epileptogenesis after brain injury may be a beneficial strategy in the management of epileptic patients.

## 3. Impact of RAS in Epilepsy

The scientific and clinical relevance of the RAS in cardiovascular hemostasis has been well established. The RAS is involved in blood pressure regulation, extracellular volume homeostasis, vascular resistance, and renal function. The system consists of several components, including the renin, angiotensinogen, several forms of angiotensin-converting enzymes and peptidases, and various receptors at many body-control levels. Stimulation of the RAS has an impact on vascular contractility and inflammation, collagen vascular deposition, oxidative stress, cellular modulation, and cardiac remodeling. Agents acting in this system primarily operate for cardiovascular disease, and belong to the three main drug classes of 1) angiotensin-converting enzyme (ACE) inhibitors; angiotensin II receptor blockers (ARBs, sartans); and direct renin inhibitors, such as aliskiren [72].

Recent studies have advanced RAS research, further clarifying its role and its involvement in brain physiology. Angiotensin peptides have been implicated in the control of seizures, during which they also act as neurotransmitters and neuromodulators in neuronal pathways, including the hypothalamus and forebrain; however, this balance may become impaired where neurological disorders are present [73].

The localization of the renin receptor in brain neurons and neural differentiation has clarified the mechanism of the RAS in brain-induced neuropathologies such as epilepsy. Renin and prorenin bind to the dimerized (pro)renin receptor, activating the mitogen-activated protein kinases (MAPKs) and extracellular signal-regulated kinase 1/2 (ERK1/2) signaling, which results in actin polymerization, the synthesis of profibrotic genes, TGF-β, plasminogen-activated inhibitor, collagen-I, and fibronectin, activation of the angiotensin II-dependent pathway and angiotensin receptor 1 (ATR1), which further results in end-organ damage [74]. This is supported by the beneficial role of the renin receptor in appropriate neural differentiation in the brain, suggesting that angiotensin peptide generation in the brain is also essential to the physiological conditions [75].

However, certain studies have shown this correlation of the RAS or renin receptor mutations to be an early step in angiotensin I synthesis, resulting in mental retardation and epilepsy [76]. The RAS has been shown to increase in the cortex zones and hippocampi of patients with TLE. An increase in ATR1 receptor expression has been determined along with mRNA expression in both zones, and with ATR2 only in the hippocampus, without changes in mRNA level occurring, showing the implication of the RAS in the pathophysiology of mesial temporal sclerosis and epileptogenesis [77].

It is also known that the main angiotensin receptors (ATR1, ATR2, and ATR4) are expressed differently in the following areas: postrema, amygdala, caudate-putamen, cerebellum, cortex, globus pallidus, hippocampus, lateral and medial septal areas, mesencephalon and thalamus [78]. ATR2, however, is expressed more in the hypothalamus, thalamus, brainstem nuclei and motor- and learning-associated areas, and its expression increases in pathological conditions, such as neuronal and vascular injury [79].

RAS involvement in epilepsy pathology is associated with the hyperactivation of Ang II/ATR1 and ACE signaling in astrocytes, oligodendrocytes and microglia, and is induced by an increase in proinflammatory cytokines, macrophage activation, oxidative stress, and BBB dysfunction. In epilepsy, Ang II causes the seizure threshold to be surpassed in different epileptic experimental models, such as pentylenetetrazol (PTZ), bicuculine, or picrotoxin. There is also a compensatory mechanism due to the upregulation of Ang 1–7, and an increased expression of ATR2 and ATR4.

Because of this, recent studies have focused on the cerebroprotective (anti-inflammatory) effects of ACE 2/Ang (1–7)/almandine through MAS receptors (MasRs) and Mas-related G-protein-coupled receptor (MrgDs) activation, which opposes the Ang II activation of ATR1. The activation of ATR2 has shown a positive role in cell survival, oxidative stress, inflammation and the remyelination process, which contribute to neuroprotective effects, ATR4 activation has been found to ameliorate the dysfunction in neuron metabolism, and provide neuroprotective effects through dopamine and serotonin release in the hippocampus [80,81].

A more detailed analysis has shown that the expression of Ang 1–7 is modified in various phases of induced animal epilepsy, with increased expression in acute and silent phases, rather than chronic phases, with activation of the MasR during the silent period [82]. The induced epilepsy in this study was characterized by an increased expression of Ang II and a reduced expression of ACE in all phases. This suggests an alternative pathway through the tonin enzyme, which acts on the conversion of angiotensinogen and Ang I to Ang II [73,82]. The Ang II–IV neuropeptides have been shown to have potentially anticonvulsive effects in different models of induced seizures [83]. This demonstrates a positive interaction with other neurotransmitters, such as adenosine receptors in the PTZ seizure model [84,85].

The beneficial effects of Ang II and IV in neuroplasticity, cognitive function and epilepsy, through the activation of ATR2 and ATR4 with appropriate ligands in animal models, have been acknowledged, creating space for the development of novel therapeutic targets for the treatment of epilepsy and memory impairment [86]. Furthermore, a long-term intracerebroventricular (ICV) infusion with Ang II in kainate-induced status epilepticus results in decreased latency in the onset of first spontaneous motor seizures (SMS) and increased SMS frequency, although it has also shown a neuroprotective role in neural damage in the hippocampus and this infusion exacerbated epileptogenesis through kainate-induced hyperactivity, and induced depressive behavior [87]. There have been some positive findings for other neuropeptides, including apelin, as an endogenous ligand of angiotensin receptor-like 1 (APJ) in the PTZ-induced epilepsy model, and the reduction of APJ with apelin-13 in the PTZ group has indicated anticonvulsive (seizure-inhibition threshold, tonic–clonic latency) and neuroprotective properties that further enhance the role of angiotensinogen pathway targeting in epileptogenesis [88].

Taking all of these findings into consideration, progress is being made in investigating new, targeted therapies, such as angiotensin drugs alone, or in combination with other drugs, including neuroprotective agents, that can provide a rational strategy for the treatment of neurodegenerative disorders, including epilepsy. ACEi and ARBs are considered to be novel therapeutic strategies for normalizing pathological conditions, such as neurological disease and cognitive dysfunction, through the CNS angiotensin pathway. ATR2 stimulation through Ang II or other selective agonists, after treatment with ARBs, is another possible therapeutic pathway for neurological disorders and damage. However, there remains a need to further clarify the beneficial effects of RAS-acting agents in the neuronal pathway so as to develop a new therapy for targeting epilepsy pathophysiology and its treatment [89].

## 4. Targeting RAS in Epilepsy Treatment

New insight into RAS modulation provides evidence for it role in the management of epileptogenesis. The direct inhibition of RAS with beta-blockers and renin inhibitors might have therapeutic implications for the pathological development of epilepsy, as well as end-organ damage, with the potential for contraindication, and also in epileptogenesis due to the reduction in renin, which has protective effects in neuronal differentiation [90]. However, beta-blockers such as propranolol, metoprolol, timolol, and pindolol have shown inhibition of epileptiform activity, with an add-on effect related to AEDs in induced epileptic models [91].

The renin inhibitor aliskiren has shown neuroprotective effects in rat cortical neuronal toxicity induced by an amyloid beta-peptide through renin release reduction from the amyloid beta which, in turn, may also be implicated in epilepsy and cognitive impairment [92]. The improvement in cognitive impairment with aliskiren has also been demonstrated in experimental models, suggesting a further positive role in epilepsy comorbidities [93].

Aliskiren has proved to have positive effects in ischemic stroke and in improving neurological outcomes following stroke, which is commonly related to acquired epilepsy. It displays antiapoptotic activity, improved neurological deficits and infarcted volume through activation of the phosphoinositide 3 kinase (PI3K/Akt) pathway, beta cell lymphoma 2 (Bcl-2) expression, and a reduction in Bcl2-associated X protein expression [94,95]. This positive effect of renin inhibition has been confirmed in the PTZ-induced animal-seizure model that was treated with AEDs and this combination contributed to an increase in the PTZ threshold, which enhanced the protective actions of clonazepam, phenobarbital, and valproic acid in the PTZ test, offering additional benefits in memory impairment [96]. These data were also replicated in a maximal electroshock seizure (MES) model in mice, supporting the synergistically beneficial effects in anticonvulsive action, suggesting its potential involvement in epileptogenesis prevention or management [97].

## 5. Angiotensin Receptor 1 Antagonists in Epilepsy

As mentioned above, an increase in ATR1 expression in cerebrovascular disease is associated with an increased risk for epileptogenesis and inhibition of the TGF-β pathway in activated astrocytes after brain injury with ARBs. In particular, losartan use in this study may have decreased the rate of BBB breakdown, which contributes to inhibition of the epileptogenesis process, and may be a beneficial strategy in the management of epileptic patients [98].

Moreover, the ARBs were found to inhibit serum-derived albumin-induced TGF-β signaling, which affects epileptogenesis progression and the development of recurrent spontaneous seizures [99]. Hence, ATR1 inhibition is more involved in neuroinflammatory suppression, which further supports the delayed mechanism of epileptogenesis through the neuroinflammation pathway. Due to this, the increased levels of Ang II and ATR1 in the microglia in the lithium-pilocarpine-induced epilepsy model were inhibited with ARBs, including losartan, which resulted in a reduction in microglia-mediated inflammation, epileptic cognitive impairment, neural loss and other neuroprotective effects, suggesting an interesting approach in the prevention of epilepsy and its comorbidities, including cognitive function [100].

In addition, the use of other ligands, such as the selective ATR1 antagonist ZD7155 in the kainate-induced epilepsy model in hypertensive and normal rats, has been investigated to further understand the new targeted therapy for the prevention and better management of epilepsy. In this study, the selective inhibition of the ATR1 receptor antagonist inhibited hippocampal monoamine levels without showing an impact on seizure development [101]. Other studies have demonstrated that ATR1 inhibition with losartan has a beneficial effect on oxidative stress and neurotoxicity induced by status epilepticus during kainate-induced epileptogenesis in rats. In one study, the rate of seizure-free periods increased, and also showed a decreased frequency of SMS, with additional benefits in improving behavioral changes—such as impulsivity, anxiety, depression, and diurnal changes in motor activity—and exhibited neuroprotective effects in the CA1 area of the hippocampus by also lowering 5-hydroxytryptamine levels [102].

The ATR1 antagonists, including telmisartan and olmesartan, have been shown to have anticonvulsant actions toward induced MES and PTZ-induced seizures in animal models. The antiepileptic effect was dose-dependent, with higher doses of ATR1 antagonists favouring the use of more telmisartan [103]. In addition to this, a recent study has investigated whether the ICV administration of losartan reduced the development rate of both behavioral- and stimulus-induced seizures at an early stage of the amygdala kindling model [104].

Similarly, the use of the ATR1 antagonist losartan has been found to reduce status-epilepticus-induced oxidative stress in the rostral ventrolateral medulla, which impacted the improvement of baroreflex-mediated sympathetic vasomotor tonus in experimental temporal lobe status epilepticus through the inhibition of superoxide anion generation from the p47^phox^ subunit of nicotinamide adenine dinucleotide phophate oxidase, nitric oxide synthase II. This induced peroxynitrite formation, and the upregulation of brain-derived neutrophic factor (BDNF) TrkB signaling, which inhibits p47^phox^. This further highlighted the positive effects of the ATR1 antagonist in reducing the mortality associated with epilepsy [105]. This may also be supported by the positive role of losartan in hypertension in the animal epilepsy model by providing additional evidence that losartan is a useful therapeutic strategy in TLE associated with hypertension [106].

In a related study, using ACEi and ATR1 antagonists, only captopril from the ACEi group reduced PTZ-induced seizures (myoclonic convulsions) in mice, also suggesting some potential involvement of ACEi in this process [96].

## 6. Antiepileptic and RAS Inhibition Drugs

There are early reports regarding AED effects in RAS. Phenobarbital was first investigated for RAS or renin serum concentration modulation through either increasing renin activity or suppressing renin release; patients with seizure disorders and low phenobarbital levels had double the plasma renin levels compared to normal adults [107]. Later studies have shown that benzodiazepine and acetazolamide can interfere with RAS, but, in general, there is a lack of systematic data regarding the effect of AEDs on the adrenal medulla and catecholamine metabolism, due to the blood supply from the cortex to the adrenal medulla, which may be affected by cortical hormonal composition and medulla function [108].

In addition to these studies, AEDs have been found to inhibit the RAS. Such effects include diazepam activity in the ACE enzyme in rat brains under normal and stressed conditions, further clarifying the involvement of benzodiazepine in the RAS [109]. Carbamazepine has also demonstrated inhibitory action of the ACE enzyme in the hippocampus, which may be linked to the beneficial effects of RAS inhibition and its role with AEDs alone in epilepsy pathophysiology, characterized by increased RAS expression and activation [110]. Other AEDs, including valproic acid, have been shown to affect the RAS, exhibiting positive effects related to hypertension, renal damage, and dyslipidemia in nitric-oxide(NO)-deficient hypertensive rats [111].

Despite the role of AEDs in interfering with RAS, research has also been focused on investigation of the potentially positive pharmacodynamic interactions or additive effects of angiotensin and related receptor-blocker ARBs in the existing AEDs. A combination of subthreshold doses of Ang II and GABAergic drugs in PTZ seizure model suggests the potentiation for antiepileptic activity and the interaction of angiotensin and GABA receptors [112,113]. However, various studies have testified to the positive effects of the AED combination therapy with the ATR1 receptor antagonist, which is considered to be a new therapeutic opportunity in epilepsy management, although there is a need for increased care in some of the combinations.

Due to this, in a recent study, the combination of ARBs, including losartan and telmisartan, with AED tiagabine not only did not demonstrate anticonvulsant activity, but it was also shown not to play a role in the anticonvulsant activity, and was recommended for use with caution in patients with either hypertension or epilepsy due to potentially neurotoxic effects, such as impaired motor coordination [114]. In another study, the combination of the ATR1 antagonist losartan, but not telmisartan, and ACEi with gabapentin improved the antiepileptic effects due to a change in the pharmacokinetic profile, rather than affecting the pharmacodynamic response, which requires monitoring; this must be used with caution in clinical practice due to the potential development of motor impairment [115].

The positive interaction of antihypertensive drugs, including ARBs, ACEi and diuretics, was tested in combination with AED levetiracetam in a MES so as to investigate their synergistic effects in better epilepsy management. In this study, only perindopril arginate positively influenced the pharmacodynamic properties of the antiepileptics [116]. However, in a related study, the ACEi (zofenopril, fosinopril, enalapril, and captopril) was shown to have added pharmacodynamic effects on AEDs (carbamazepine, felbamate, felbamate, lamotrigine, topiramate, and valproate) in animal models with a generalized tonic–clonic seizure. The co-administration of fosinopril with valproate and lamotrigine was found to have the most dominant and favourable pharmacological response [117].

Similarly, a combination of losartan and telmisartan with older AEDs (carbamazepine, phenytoin, phenobarbital, and valproate) has been shown to influence antiepileptic activity in the MES animal model, with losartan and telmisartan differentiated in improving the anticonvulsant effect of valproate and this positive interaction was characterized by its pharmacodynamic nature, which supports this type of combination in targeting both epilepsy and hypertension [118]. This was additionally supported by the combination of an ATR1 antagonist (losartan, telmisartan) and second-generation AEDs (lamotrigine, topiramate, and oxcarbazepine), which resulted in enhanced protective anticonvulsive actions in MES-induced seizures. The combination of losartan and lamotrigine was found to have a pharmacodynamic nature [119].

Few studies have shown the potentiation of the antiepileptic effects of lamotrigine in the model of MES-induced generalized tonic–clonic seizures, by targeting RAS through the earlier inhibition of angiotensin II synthesis through enzymatic reaction inhibition, including ACEi alone and AEDs. These enhanced effects have also been related to the pharmacodynamic process [120]. Similarly, the ACEi enalapril enhanced the therapeutic effects of valproate, which has also affected the pharmacodynamics process in MES seizures [121]. The same group supported these data by showing that the other ACEi, captopril, positively affected carbamazepine and lamotrigine antiepileptic actions without showing beneficial effects with the other AEDs investigated [122].

## 7. New Perspectives in Targeting RAS MAS, ATR2, and ATR4 Receptors in Epilepsy

While there have been promising findings in RAS modulation and the prevention and management of epilepsy, there is still interest in the investigation of alternative approaches. One of the possible alternatives is the targeting of the ACE2-Ang-(1–7)-MasR, which results in an increase in NO and prostaglandins, reduces oxidative stress, and induces diuresis [123]. Many studies have demonstrated positive effects in cerebral ischemia and neuroprotection with a MasR agonist (ACE0991), which may be also associated with the beneficial effects in acquired epilepsy [124]. The role of the MAS axis in macrophage-mediated inflammation has been further clarified in animal in vivo studies, showing great potential for developing neuroinflammation and also playing a major role in vascular function and related diseases, including atherosclerosis development [125]. MasR signaling opposes the RAS microglial proinflammatory response and AngII/AT1R-mediated effects. This has beneficial effects in neuroinflammation through the modulation of macrophage polarization, migration, and T-cell activation [126].

In a recent study, AVE0991 was found to attenuate the neuroinflammation related to the ageing process through the suppression of the microglial-mediated inflammatory response [127]. In addition to this, the intranasal administration of ACE0991 in the animal study reduced oxidative stress and neural apoptosis, and improved neurobehavioral scores in brain injury after subarachnoid hemorrhage. These responses were reversed after the selective inhibition of MasR, which further increased evidence for the management of brain-related injuries [128]. Another approach in the activation of the Mas axis with the ACE2 activator diminazen aceturate has demonstrated anti-inflammatory potential through inhibition of the p38MAPK, JNK, ERK1/2, and NF-kB pathways [129]. This has been supported by previous findings, which have suggested that ACE2 overexpression attenuates neurogenic hypertension, neuroinflammation, and oxidative stress, and further supports Mas axis signaling and neuroprotective roles, which may contribute to acquired epilepsy [130].

The activation of the MasR axis with diminazen aceturate has been found to have a beneficial role in the cognitive process of neurological diseases, such as Alzheimer’s, which might be related to the improvement of neuroplasticity and cognitive function in patients with epilepsy through the activation of the PI3K/Akt pathway, AMPc, a brain-derived neurotrophic factor, nicotinic and glutaminergic receptors, and the inhibition of tau and glycogen synthase protein contents, NF-kB and TNF-alpha [131].

Moreover, the targeting of ATR2 and ATR4 has been shown to have a beneficial action in epileptogenesis. Ang II-mediated ATR1 and ATR2 receptor activity is regulated through the crosstalk activity between physiological and pathological conditions. Therefore, the ATR2 is mainly characterized by cell differentiation effects, neural regeneration, apoptosis inhibition, and neural protection in brain injury, which suggests a beneficial role in counteracting the pathophysiology of epilepsy [77]. Because of this, development of the ATR2 agonists is an interesting approach for the management of neurological disorders, including epilepsy pathophysiology and treatment outcomes. ATR2 agonists are firstly represented by nonpeptide compound 21 (C21). Several developed nonpeptide ATR2 agonists have shown anti-inflammatory effects through the inhibition of NF-kB and Janus kinase, which also results in an increase in the synthesis of 11-, 12-epoxyeicosatrienoic acid.

Similarly, Ang II analogues with a peptide structure also activate ATR2 [132]. The ATR2 agonist also has antifibrosis, vasodilatation, antiproliferation, and neuroprotection effects; increases BDNF; and inhibits neuronal apoptosis, whilst showing beneficial effects in cognitive function in ischemic brain damage after stroke [133]. The common preclinical administration of ATR2 agonists is via the ICV or systemic administration routes, which makes it difficult or unsuitable to replicate the such studies in humans due to the lower BBB permeability of the agonists. However, recent administration via the nose-to-brain route, in order to bypass the BBB, of the selective agonist ATR2 C21 in induced-ischemic-stroke rats has proved to be a good pathway for increasing brain bioavailability that may also find clinical application in human-related studies.

In this study, C21 entered the cerebral cortex within 30 minutes, and significantly reduced the cerebral infarct size, which resulted in an additional improvement in neurological scores, suggesting that this new administration pathway presents a good opportunity for a better pharmacokinetic profile, and further supports the evidence for using an ATR2 agonist in ischemic-stroke-patient neuroprotection [134]. There is also evidence that disruption of the BBB in CNS diseases allows C21 to enter the CNS and exert its central actions [132]. Moreover, a recent study has demonstrated the positive effects of C21 in post-stroke cognitive impairment, which provides further evidence regarding the neuroprotective roles that might be involved in epilepsy [135].

Despite preclinical development of the ATR2 agonist, clinical findings have previously reported the role of mutations in this receptor, and its association with the development of mental disorders and epilepsy, suggesting there are further indications to be considered for the future role of an ATR2 agonist and the potential for investigation of pharmacogenetic approaches to individualize later clinically developed agonists to further clarify its beneficial role [136]. Similarly, the ATR4 (insulin-regulated membrane aminopeptidase) agonists may be a potential target for the improvement of cognitive function in epilepsy and AED-induced memory impairment [137]. This receptor plays a role in neuroplasticity and potentiates the learning and memory pathways in the hippocampus CA1 region [138]. ATR4 has become an important target for the treatment of neuropathologies, including epilepsy [139].

**Figure 1:**
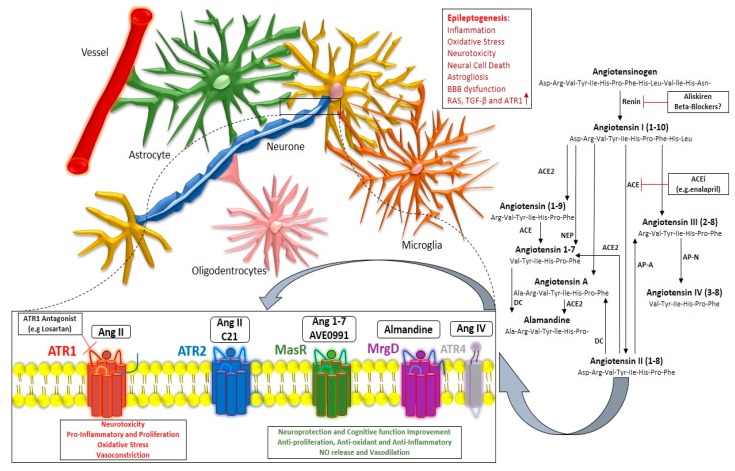
Contribution of renin–angiotensin system-mediated actions in the epileptogenesis process. Exacerbation of epilepsy pathophysiology through predominance of the Ang II/ATR1 axis, and available targeted therapeutic strategy through RAS enzyme inhibitors; ATR1 antagonists; and ATR2, MasR, MrgD, and ATR4 agonists that mediate beneficial actions in epilepsy pathophysiology and related consequences.

The anticonvulsant effects against pilocarpine-induced seizures has demonstrated the implications of neurotransmitters, including increased serotonin and dopamine in the hippocampus, somatostatin receptor-2 activation and the inhibition of insulin-regulated aminopeptidase [140]. Bearing this in mind, there has been great interest in developing novel peptide analogues for ATR4 due to their role in cognitive function and epilepsy [86]. The involvement of RAS in epileptogenesis in neurons, microglia, astrocytes, and BBB, and targeted therapeutic approaches through enzyme inhibition, ARBs and selective ATR2, ATR4, MrgD, and MasRs is depicted in Figure 1.

## 8. RAS Genetic Studies in Epilepsy

There has been some interest in investigating the genetic associations and pharmacogenetic approaches in the RAS pathway in other, related pathologies, such as hypertension and neurological disorders, cognitive function, stroke and Alzheimer’s. Most studies have involved renin, angiotensinogen, ACE, ACE2, ATR1, and ATR2 [141,142,143,144,145]. However, there have been few reports on the association of the renin receptor ACE and ATR1 in relation to mental disorders and epilepsy [76,110,136], suggesting a need for pharmacogenetic and genetic association studies targeting the RAS pathway in epilepsy.

As pharmacogenetic approaches in the individualization of epilepsy treatment continue to develop, RAS may be a potential pathway for further investigation, and for consideration as a new approach to personalizing epilepsy treatment. This may further contribute to the elucidation of pharmacoresistence.

## 9. Conclusions

In understanding that epilepsy pathophysiology is complex and not definitive, there are many pathways that can contribute to the epileptogenic mechanism. The epilepsy pathological process in neurons, astrocytes, and glial cells is primarily affected by inflammation, oxidative stress, neurotoxicity, neural cell death, and BBB dysfunction, which contribute to an imbalance in neurotransmission, resulting in aberrant neuronal excitability and epileptogenesis.

Therefore, the involvement of the RAS in the process of epileptogenesis and targeted therapy approaches presents a new strategy for the better management of epilepsy. Using RAS pathway inhibitors or RAS receptor-selective antagonists or agonists alone, or in combination with current AEDs, has provided new pharmacological insights that may further contribute to better epilepsy management and treatment outcomes.

There have been numerous preclinical background studies, as there is an increasing interest in progressive research to highlight synergistic pharmacodynamic actions with current AEDs, clinical and pharmacogenetic studies for RAS and epilepsy management, and the targeting of drugs to translate them into future clinical implications.
